# Natural ageing process accelerates the release of Ag from functional textile in various exposure scenarios

**DOI:** 10.1038/srep37314

**Published:** 2016-11-21

**Authors:** Dahu Ding, Lulu Chen, Shaowei Dong, Hao Cai, Jifei Chen, Canlan Jiang, Tianming Cai

**Affiliations:** 1 College of Resources and Environmental Sciences, Nanjing Agricultural University, Nanjing 210095, China

## Abstract

Natural ageing process occurs throughout the life cycle of textile products, which may possess influences on the release behavior of additives such as silver nanoparticles (Ag NPs). In this study, we assessed the releasability of Ag NPs from a Ag NPs functionalized textile in five different exposure scenarios (i.e. tap water (TW), pond water (PW), rain water (RW), artificial sweat (AS), and detergent solution (DS) along with deionized water (DW) as reference), which were very likely to occur throughout the life cycle of the textile. For the pristine textile, although the most remarkable release was found in DW (6–15 μg Ag/g textile), the highest release rate was found in RW (around 7 μg Ag/(g textile·h)). After ageing treatment, the total released Ag could be increased by 75.7~386.0% in DW, AS and DS. Morphological analysis clearly showed that the Ag NPs were isolated from the surface of the textile fibre due to the ageing treatment. This study provides useful information for risk assessment of nano-enhanced textile products.

Silver nanoparticle (Ag NP) is a promising engineered nanomaterials (ENM) for textile industry due to its antimicrobial, antifungal, and partially antiviral properties^1^. For example, the estimated production rate of Ag NPs in Europe was in the range of 0.6–55 t/year^2^. And the maximum amount of biocidal Ag used in textile products was estimated to be 9–45 t/year globally^3^,^4^. On the other hand, it was well believed that ENM were inevitably released into the environment during the production, use and disposal of the nano-enhanced products. Specifically, the textiles at disposal phase would lose up to 10% of the ENM weight through abrasion during washing and usage^5^. Though the predicted environmental concentration (PEC) of Ag NPs in the aquatic environment had been reported to be 0.03 μg/L^6^, its strong toxicity towards aquatic organisms^7^ still called for a cautious risk assessment. As a result, a great deal of attempts had been made to investigate the potential risks of the Ag NPs textiles derived commercial products such as socks^8^,^9^, shirts^10^, trousers^10^ and medical masks^11^. For example, Benn and Westerhoff^8^ found that as much as 650 μg silver (both colloidal and ionic forms) was released into 500 mL distilled water from socks. Subsequently, in order to mimic the more realistic conditions, Geranio *et al.* 9 updated the experimental setting and indicated that the majority of the released silver was in the size fraction >450 nm during the washing process. Also, the Ag NPs could migrate into sweat when the textiles directly contact with the skin^10^,^12^. All these studies confirmed the phenomenon that silver could be released from textiles due to chemical mobilization or physical stress.

On the other hand, the release and transformation processes are complex and dependent on the solution chemistry, redox environment, and particle specific characteristics^13^,^14^,^15^. For example, ultrapure water was found to result in a much more significant release than tap water^8^. The bleaching agents could greatly accelerate the dissolution of silver and transform Ag^+^ to AgCl^16^. Silver might be released as single particles, agglomerates, embedded in the matrix, or as dissolved ions^15^,^17^,^18^,^19^,^20^. For instance, larger fractions of dissolved silver (Ag-chloro complexes) rather than particulate form were found in sweat^10^.

Although various release scenarios had been considered, the scientific understanding was still hampered by the narrow scope of previous works. A comparative study among the different exposure media throughout the product life cycle (use/wear, washing and final disposal) was in an urgent demand. Particularly, the textile products would be inevitably aged during their life cycle. Ageing process means the chemical compositions, physical properties, and structure of polymer materials (fibre, plastic, and rubber) will be changed during their usage affected by the light, heat, microorganisms, and other environmental factors. Also, the attached ENM on the functionalized textiles might be changed^21^. Therefore, it was hypothesized that the ageing process would probably alter the subsequent release behavior of Ag NPs. From an environmental point of view, it is very important to unravel this phenomenon. However, to the best of our knowledge, little information is available up to the present^22^.

The aim of this work was to investigate the effect of ageing treatment on static release behaviors of Ag NPs from a commercially available Ag NPs functionalized textile during the product life cycle. Specifically, rain water (RW) and artificial sweat (AS) were prepared to study the exposure scenario during the use/wear of the textile products. Tap water (TW) and detergent solution (DS) were used to investigate the release behavior during the washing process. Also, pond water (PW) was collected and used to reveal the release behavior during the possible disposal of the textile products. In addition, deionized water (DW) served as a reference throughout the tests. Particle fraction of silver <450 nm and the dissolved fractions in these solutions were analyzed by using inductively coupled plasma-optical emission spectroscopy (ICP-OES), transmission electron microscope (TEM), and energy dispersive X-ray spectroscopy (EDX).

## Materials and Methods

### Materials

All the chemicals were of AR grade unless otherwise stated. DW and ultrapure water (18.2 MΩ cm) were obtained from a laboratory water purification system. Commercially available Ag NPs functionalized textile was purchased from JLSUN High-Tech Co., Ltd. (Beijing). The Ag NPs were coated on the fibre through multi-target vacuum magnetron sputtering technique. An edge zone of 1 ± 0.5 cm of the textile was discarded and not used in the experiments. The Ag contents in the triplicates textile samples were quantified by ICP-OES (VARIAN 720 ICP-OES) (with detection limit of 5 mg/kg) after complete digestion. Details of the digestion procedure are available in supporting information. Silver plasma standard solution (1,000 μg/mL Ag in 1 M HNO_3_) obtained from National Standard Substances Center, China was stored in the dark (prevent photoreduction) and used for calibration. The ICP results showed that the textile samples contained 53,909 ± 14 mg Ag/kg textile, much higher than most of the previously investigated textiles^8^,^9^,^10^,^17^,^19^ except a medical mask and cloth (containing 230~270 mg Ag/g product)^11^.

TW was obtained in the laboratory and used without any treatment. PW was collected from a pond located in Weigang campus of Nanjing Agricultural University, Nanjing, China. After collection, the PW was filtrated with filter papers (Whatman) to remove the suspended solids and algae. RW was collected during a rainfall occurred in August, 2015. Similar to PW, the collected RW was filtrated and stored at 4 °C before use. AS was prepared according to the method 125–2004 “Colorfastness to perspiration and light” of American Association of Textile Chemists and Colorists (AATCC) with slight modification. Typically, 0.25 g L-histidine monohydrochloride, 10 g sodium chloride, 1 g lactic acid, and 5 g disodium hydrogen phosphate dodecahydrate were thoroughly dissolved in 1 L DW. The AS was stored at 4 °C prior to use. The DS was prepared by dissolving a commercial liquid detergent purchased from the local market into TW at a ratio of 1:1000 (v/v) according to the product instruction. The commercial liquid detergent contained surfactant, detergent builder, and bleaching agent. The properties of the media used in this studyare given in Table 1.

### Artificial accelerated ageing treatment (AAAT) of textile

The coarse AAAT was conducted according to the method “Weather resistance: UV light and moisture exposure” recommended by AATCC (186–2006) with slight modifications. An UV ageing lamp (UVA-340, 40 W, 1200 mm) was utilized as the UV light source. The textile samples cut into long strips (L × W = 30 × 5 cm^2^) were placed in parallel where was about 2 cm away from the lamp. The temperature and humidity were maintained respectively at approximately 25 ± 2 °C and 60% throughout the experiment. Surface morphology of textiles before and after the experiments were observed by using an SEM (Zeiss EVO 10) equipped with an EDX attachment (Oxford). To make the results more comparable, two ageing treatment durations (12 h and 48 h) were applied along with the no ageing treatment as a reference in this study.

### Release of silver from textile

Approximately 0.5 g textile sample (containing ~27 mg Ag) was added into 10 mL of each aqueous solution in the 15-mL polypropylene vial which was under gentle agitation (150 rpm) in a vapor-bathing shaker (HZ-9310KB). All the vials were washed with 10% HNO_3_, rinsed with DW for 5 times and dried prior to use. The exposure duration and temperature were dependent on the exposure scenario. Specifically, the exposure duration was 15 d for DW and PW, 1 h for TW, RW, AS and DS. In addition to ambient temperature (25 °C), relatively low (15 °C) and high (35 °C) temperatures were sometimes employed to mimic extreme conditions. For instance, temperatures of the media in wintertime could be around 15 °C. Meanwhile, the temperatures of the PW (surface layer) and sweat could exceed 30 °C in summer. All the release experiments were conducted under room light to mimic the natural exposure conditions. Noting that, the results were expressed not only by release amount (μg/g) but also release rate (μg/(g·h)) because of the distinct release durations among different scenarios.

### Separation of Ag NPs and ionic silver species

The separation and analysis of silver species were carried out according to a previous study^10^. Typically, the collected aqueous samples were filtrated through polyether sulfone syringe filters (ANPEL, Shanghai) with pore sizes of 0.45 μm and 0.22 μm successively. And particulates <450 nm and <220 nm were determined as the fractions in the filtrates passed through 0.45 μm and 0.22 μm membranes, respectively. The filtrate was acidified with HNO_3_ before ICP analysis. Finally, in order to determine the fraction of silver in dissolved form, the remaining filtrate was further fractionated by filtration with Amicon Ultra centrifugal filters with a cutoff of 30 kDa (Millipore, 30 000 MWCO, centrifugation for 10 min at 8,000 rpm). Typically, for the acidification process, 1.5 mL of the filtrate/centrifuged solution was mixed with 0.9 mL of ultrapure water and 0.6 mL of HNO_3_ (65%). All the acidified samples were refrigerated at 4 °C prior to ICP-OES (Agilent 710 ICP-OES) analysis. All data were presented with the standard deviation of at least two independent samples.

### TEM/EDX analysis

Typically, approximately 1 mL aqueous sample was centrifuged (Eppendorf centrifuge 5418) at 12,000 rpm for 5 min and 0.5 mL supernatant was discarded. Then another 0.5 mL fresh sample was added and the centrifugation was repeated. After that 0.5 mL supernatant was discarded and the remaining mixture was ultrasonically dispersed for 1 min in an ultrasonic cleaner (HS-2060A, 40 kHz). Finally, drops of the dispersion were evaporated on the TEM grid (copper with carbon coating) for analysis. TEM/EDX analysis was performed on a TEM (Tecnai G2, F20, FEI) attached with an EDX system (EDAX). The TEM was operated at an acceleration voltage of 200 kV.

### Other analytical methods

All solution pHs were measured with a pH meter (Leici) and given in Table 1. The UV spectra (200–600 nm) were recorded by using a Cary 50 spectrophotometer (VARIAN) and given in Figure S1. In order to determine the organic and inorganic contents in the samples, dissolved organic carbon (DOC detected by Elementar vario TOC meter) and salinity (only for TW, PW, and RW) analysis were also conducted respectively. The three-dimension excitation emission matrix (EEM) fluorescence spectrophotometer (Cary Eclipse, VARIAN) was adopted to characterize the dissolved organic matters (DOM) in PW and DS before and after exposure experiments. Detailed procedures of these tests were available in supporting information. Besides, an Agilent 710 ICP-OES was used for the detection of the metal ions. The limit of detection (LOD) of the ICP was estimated by measuring the blank samples for 10 times successively and was 3 times of the relative standard deviation (RSD). The recovery tests indicated that the recoveries were 95–104%, which were quite satisfactory. The concentrations of Ag^+^ in these acidified media (background) were all below the detection limit of the ICP-OES (data not shown). Chloride is an important ion that may lead to the formation of AgCl precipitates, and it was measured by an ICS 900 ion chromatograph (Thermo) in the current study. Dilution was sometimes necessary to ensure measurement accuracy.

## Results and Discussion

### Morphological analysis of the textiles

**Figure 1:**
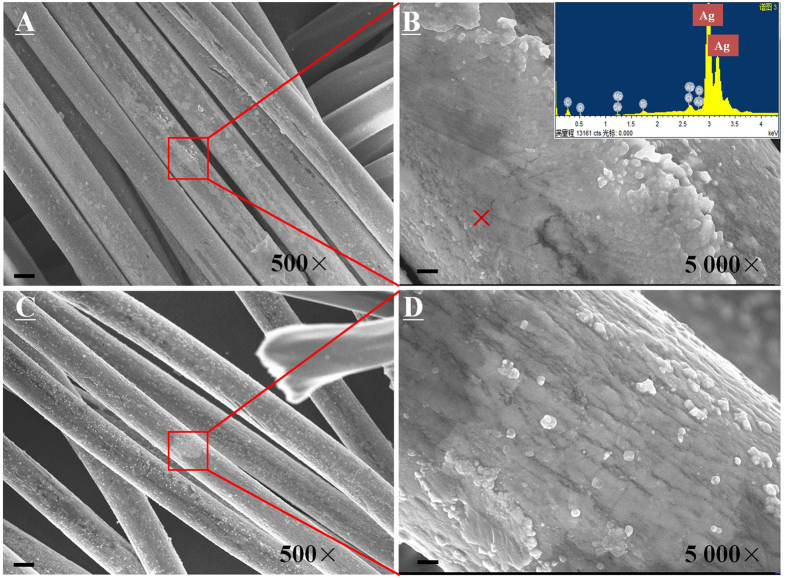
Representative SEM images of pristine ((**A**) 500×, ((**B**) 5,000×) and aged (48 h) textile ((**C**) 500×, ((**D**) 5,000×). (Inset shows the EDX pattern of the marked point in Panel B, scale bar in **A** and **C** is 10 μm, in **B** and **D** is 1 μm).

As shown in Fig. 1, a distinct fibre texture in the textile sample was observed. From Panel B, it could be seen that the surface of every single fibre was covered by a compact film. Combining with the EDX analysis, it was clear that the film was mainly consisted of silver. In addition, the film was incorporated well with the fibre, indicating the Ag NPs were tightly adhered onto the fibres. However, after ageing for 48 h, as shown in Panel C, the surface structure significantly changed. Numerous nanoparticles were detached from the film. Elemental analysis confirmed that the isolated nanoparticles were Ag NPs. Meanwhile, the smooth film was wrinkled to some extent after the ageing treatment (Fig. 1D). As discussed, the UV light would bring structural damage to the textile during the ageing treatment. Consequently, the structural variation was probably due to the damage of the capping agent between Ag NPs and the fibre. It was believed that the isolated Ag NPs would enhance their releasability. After exposure, as given in Figure S2, Ag NPs and agglomerates were still observed clearly on the fibre surface.

### Release of silver from pristine textile

**Figure 2:**
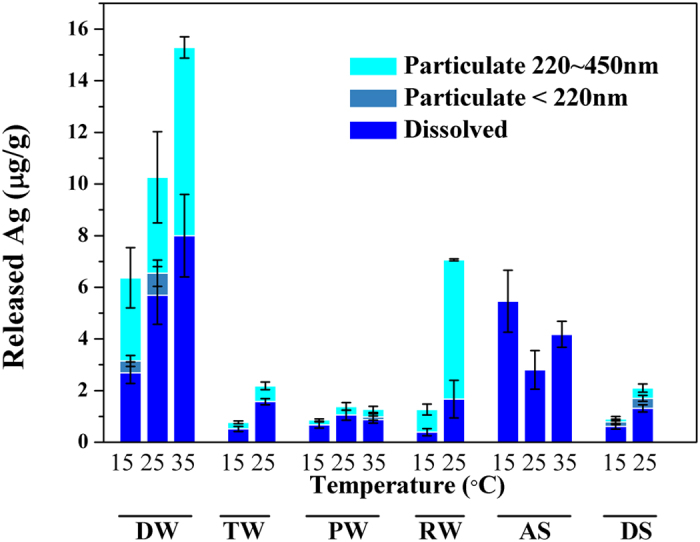
The release behavior of the nano-Ag functionalized textile in different media under different temperatures. (Error bars were generated among the results of two replicates, the exposure duration was 15 d for DW and PW while 1 h for TW, RW, AS, and DS).

As shown in Fig. 2, the amount of Ag released varied significantly in different exposure scenarios, which was not beyond the expectation when taking the distinct solution chemistry (Table 1) into consideration^14^. The most remarkable release was detected in the DW among all the tested media. Similarly, Benn *et al.*^8^ also found that much more silver was released from Ag NPs socks in ultrapure water than tap water. This result indicated that inorganic salts (salinity), organic matters, or other substances in the matrix might inhibit silver from releasing. However, the release rate in DW (around 0.017~0.042 μg/(g·h)) was relatively small considering the long release duration. Low temperature was unfavorable for the release whereas high temperatures accelerated the release process. In addition, over 6 μg Ag/g textile was released in RW at 25 °C, indicating the high potential risk of the textile during the rainfall. On the other hand, the most insignificant release was observed in PW among the tested media. The release rate was even as low as 0.003 μg/(g·h). There was no obvious variation between DS and TW scenarios, though the bleaching agents existed in DS had been demonstrated to accelerate the dissolution of silver (oxidate Ag to Ag^+^) and to transform Ag^+^ to AgCl^16^. A possible reason was that the interactions between the bleaching agents and other components in the DS might lower the amount of available agents.

Overall, approximately 0.8–15 μg Ag/g textile was released into the tested media, which was relatively low when compared to other studies^8^,^9^,^10^,^11^. Considering the relatively high silver content in the current textile (53 909 ± 14 mg Ag/kg textile), the release amount was only 0.001–0.03% of the initial silver content. This value was much lower than those reported in most previous studies (3–14% in AS or 20–30% in washing solutions)^8^,^9^,^10^,^11^,^12^,^17^ except that indicated by Yan *et al.* (0.02–0.05% in AS)^23^, whose textile sample also contained an extremely high metallic silver content of 17% (18% in current study).

The manufacturing process had been identified to play a key role in controlling the release of silver from textiles^8^. This might be a possible reason for the low Ag release. In Yan’s study^23^, the textile was used as an “electromagnetic wave repellent” textile, which was also a potential application of the textile investigated in this study from the description by the manufacturer. Consequently, the release amounts were comparable between these two textile products probably due to the similar manufacturing process. In addition, the physical stress had been applied in many works and it might play a dominant role during the release process^9^,^10^. This might be another reason for the relatively low release amount of Ag in the current study.

The species of silver released from textile in different media also varied significantly. The dissolved form dominated the total released silver in all the tested media except RW. This result was reasonable because the ubiquitous dissolution and oxidation of the Ag NPs. Over 50% of the silver was detected as dissolved form in DW, partially in agreement with the finding of Benn and Westerhoff who claimed that about 70–90% of released silver from an Ag NPs functionalized sock was in ionic form in distilled water^8^. However, Ag NPs could also be formed from dissolved silver by many substances such as reduced humic acids^24^,^25^, dissolved organic matters^26^, superoxide^27^, extracellular polymeric sbstances (EPS)^28^ under environmental conditions, which was possible to occur in RW.

Noticeably, only dissolved form was observed in AS. A previous study indicated that the released silver existed as dissolved Ag-chloro complexes due to the high chloride concentration in AS^10^. In addition, most particulate Ag NPs were in large size (220~450 nm), signaling the aggregation of Ag NPs in the media.

### Release of silver from aged textile

**Figure 3:**
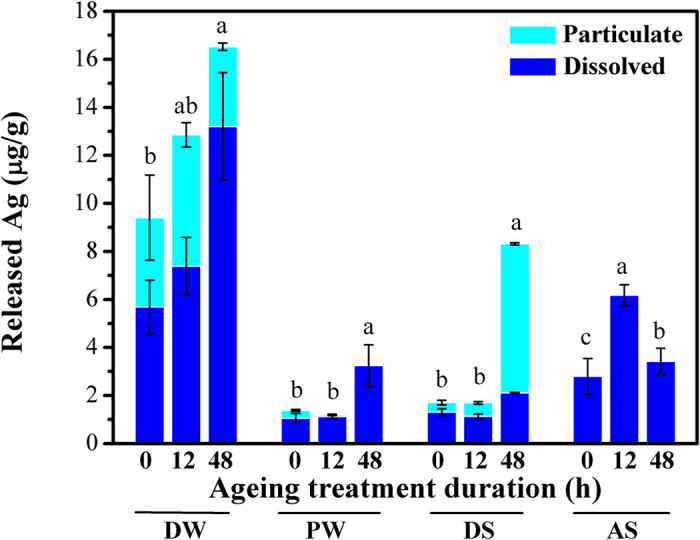
The release behavior of textiles with and without ageing treatment in different media under 25 °C (the different letters shown above the column indicated the release amount was significantly changed, p < 0.05).

As shown in Fig. 3, the released silver was significantly enhanced after ageing for 48 h, probably due to the damage of Ag NPs containing composite film (Fig. 1). Nevertheless, the enhancement was not significant for the textile aged for 12 h, indicating that ageing time played an important role during the release.

Similar to the pristine textile, the highest amount of released silver was detected in DW though the release rate was relatively low (0.047 μg/(g·h)). In addition, the released amount exhibited a strong positive relationship with ageing time in DW. On the other hand, the release process in PW was relatively weak when comparing with those in DW, AS, and DS. The total released silver increased by approximately 45% in PW when after 48 h of ageing experiment, in contrast to that of 75.7~386.0% observed with other media. These results clearly indicated that the ageing treatment could increase the chemical mobility of Ag species on the functionalized textiles.

Moreover, a dramatic difference in released species of silver was discerned among these media. In PW and AS, almost all the released silver was in dissolved form no matter from pristine or aged textile. Both particulate and dissolved forms of silver were observed in DW and DS. Dissolved form dominated the released silver in DW, which was not affected by the ageing treatment. However, the speciation of silver in DS from aged textile largely varied with that from pristine textile. After ageing treatment, the particulate silver was greatly released while the dissolved form was relatively stable. The phenomenon might indicate that the release mechanism was significantly changed due to the ageing treatment. The relevant transformation of silver species and formation of new particles would be the possible reason for the phenomenon^15^.

### TEM/EDX characterization of the released Ag NPs

**Figure 4:**
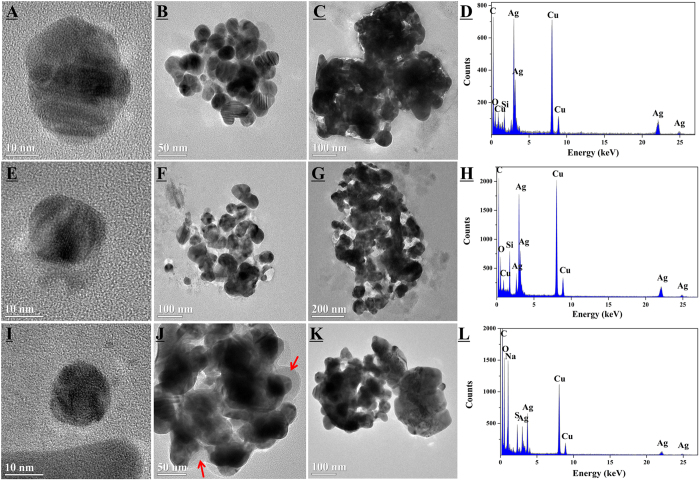
Representative TEM images and EDX spectrum of Ag NPs released from pristine textile in DW (**A**–**D**) and RW (**E**–**H**), and from aged (48 h) textile in DS (**I**–**L**) under 25 °C. (The red arrows in Panel J showed the released Ag NPs were coated with a thin film).

In order to confirm the presence of Ag NPs in the solutions, TEM/EDX analysis was conducted with DW, RW, and DS solutions after the exposure. As shown in Panel A to C in Fig. 4, some single nanoparticles and their aggregates with different sizes were clearly observed. According to the corresponding EDX patterns, distinct Ag peaks indicated the presence of metallic Ag NPs.

As known, besides Ag NPs (directly release from the textile), silver could also occur in particulate form as AgCl^17^, Ag_2_O^29^, and Ag_2_S^30^ (transformed from Ag^+^ or Ag NPs) in different aquatic environment. AgCl usually occurred due to the precipitation of released Ag^+^ and Cl^-^ in the matrix. As indicated in Table 1, the concentration of Cl^-^ in RW was 5.58 mg/L, which would be able to precipitate approximately 17 mg/L of Ag^+^ according to stoichiometry calculation. All the dissolved silver would have been precipitated as AgCl if they were occurred in Ag^+^ in this case. However, no Cl signal was observed in any of the representative EDX spectrum as shown in Fig. 4, suggesting no AgCl in the selected area. A similar result that no Cl peak was found in the EDX pattern has also been reported previously^31^. However, the missing of Cl signal might also be ascribed to the preparation of the TEM sample (ultrasonic treatment) and the area selected for the EDX analysis. Therefore, it was difficult to arbitrarily exclude the occurrence of AgCl and more technique would be necessary to confirm the phenomenon. Meanwhile, the observed O signals might indicate the presence of Ag_2_O. As well known, Ag NPs could be rapidly oxidized when being exposed to the atmosphere^32^ and thus, it was possible that the rim of the particles consisted of Ag_2_O. On the other hand, the relatively strong O signal in Panel L was probably attributable to the organics containing O binding with Ag NPs in DS^31^. As demonstrated by the DOC values (Table 1), DS contained more DOM than DW and RW. This was probably the reason why the C and O signals in Panel L were much stronger than the other two EDX spectrums. In addition, distinct Na and S peaks were observed in the Panel L. The Na peak might be attributed to the Na-containing chemicals in detergent adsorbed onto the surface of Ag NPs. The origin of the S signal remained ambiguous. One possible origin was the DOM containing S adsorbed by Ag NPs. The other possible origin was the Ag_2_S or other Ag/S particles transformed from Ag NPs^29^,^30^.

As indicated by the red arrows in Panel J (Fig. 4), some Ag NPs were coated with a thin film in DS. The film coated Ag NPs might be originated from the textile directly or due to the subsequent interactions between Ag NPs and some components in DS such as capping agents^15^. The former hypothesis could be ruled out because these film-like substances were not clearly observed in DW and RW. Probably due to the protection of the “shell”, the dissolution of Ag NPs was inhibited and thus the particulate form dominated the released silver (Fig. 3). On the other hand, as proved in many previous works, Ag NPs could interact with DOM in natural aquatic environments and thus alter their aggregation and stability^33^,^34^,^35^. For example, NOM was found to be adsorbed/coated onto the surface of Ag NPs, resulting in enhanced stabilization of the Ag NPs suspensions^33^,^35^. Suwannee River humic acids (SRHA) caused a partial disaggregation of Ag NP aggregates by nanoscale film formation^34^. Taking into account the above results and the relatively high DOC content in DS, it was speculated that some interactions might occur between Ag NPs and DOM in DS.

In order to verify this speculation, three-dimension excitation emission matrix (EEM) fluorescence spectroscopy was used. According to Table 1, PW and DS contained relatively high concentrations of DOM and therefore were selected for the EEM analysis. As shown in Figure S3, the EEM spectra of PW were similar before and after exposure (Panel A and B). Two peaks located in region I and II were identified, respectively. However, the spectra of DS were significantly altered. A distinct peak in region V disappeared while a peak in region II enhanced. This phenomenon clearly indicates that the dissolved silver complexes or Ag NPs interacted with DOM along with the release process^36^. In general, the peaks at region V were related to humic acid-like organics, while the peaks at region I and II were related to simple aromatic proteins such as tyrosine^37^. It could be deduced that humic acid-like organics had great potential to bind with dissolved silver complexes or Ag NPs.

## Conclusion

In summary, the textile functionalized with Ag NPs exhibited diverse characteristics regarding silver release in different media. The highest release amount was observed in DW while the highest release rate was found in RW. Both dissolved and particulate forms were identified in all the tested media except AS (only dissolved silver detected). In addition, the release process will be greatly accelerated with the natural ageing of the textile products. The most significant enhancement of the silver released after ageing treatment was found in DS, especially for the particulate fraction. The DOM in media might interact with silver species along with the release process. More importantly, our results suggest that the matrix effects should be taken into consideration for the comprehensive risk assessment of a nano-enhanced product in practice.

## Additional Information

**How to cite this article**: Ding, D. *et al.* Natural ageing process accelerates the release of Ag from functional textile in various exposure scenarios. *Sci. Rep.* **6**, 37314; doi: 10.1038/srep37314 (2016).

**Publisher’s note:** Springer Nature remains neutral with regard to jurisdictional claims in published maps and institutional affiliations.

## Supplementary Material

Supplementary Information

## Figures and Tables

**Table 1:** Properties of exposure media investigated in this study.

Item	TW	PW	RW	AS	DS
DOC (mg/L)	4.7	23.0	21.3	484.4	54.7
DIC^a^ (mg/L)	17.2	28.6	3.3	0	16.8
Salinity^b^ (mg/L)	75 ± 11.8	175 ± 11.8	120 ± 8.5	—	—
pH	6.7	7.1	6.8	6.2	5.8
Cl^−^ (mg/L)	15.6	42.9	5.6	6629.7	—
NO_3_^−^ (mg/L)	6.5	0.2	44.4	8.6	—
SO_4_^2−^ (mg/L)	31.6	49.9	24.8	7.0	—
K^+^ (mg/L)	3.6	8.2	2.8	46.6	5.9
Na^+^ (mg/L)	8.7	41.7	ND	6959.4	14.0
Ca^2+^ (mg/L)	30.9	28.2	22.3	ND	25.4
Mg^2+^ (mg/L)	12.6	31.6	1.7	ND	12.2

TW, tap water; PW, pond water; RW, rain water; AS, artificial sweat; DS, detergent solution. ND, not detectable (below detection limit).

^a^Dissolved inorganic carbon.

^b^According to the standard method, the salinity measurement is only for natural waters. —No measurement.
