# Supplementary material for: Natural ageing process accelerates the release of Ag from functional textile in various exposure scenarios
Source: Sci Rep. 2016 Nov 21;6:37314. doi: 10.1038/srep37314 (PMC5116759; doi:10.1038/srep37314)
Supplement: Supplementary Information [file srep37314-s1.doc]

Supplementary information

**Natural ageing process accelerates the release of Ag from functional textile in various exposure scenarios**

Dahu Ding, Lulu Chen, Shaowei Dong, Hao Cai, Jifei Chen, Canlan Jiang, and Tianming Cai*

College of Resources and Environmental Sciences, Nanjing Agricultural University, Nanjing 210095, China

Number of pages: 4

Number of figures: 3

Number of tables: 1

**MATERIALS AND METHODS**

**Digestion method**. Approximate 0.1 g textile sample was weighed and added into 50 mL tubes. Then, 20 mL *aqua regia* was added into the tube and placed into a graphite heating apparatus. The sample was heated at 200 °C until completely digested. The cooled mixture was transferred into 50 mL volumetric flask and diluted. Finally, the concentration of silver was determined by using ICP-OES (VARIAN 720 ICP-OES).

**TOC test**. The DOC and DIC concentration was measured by using an Elementar Vario TOC. All aqueous samples were passed through a 0.45 µm filter prior to the test. The samples were diluted with DW to ensure the accuracy of the results. Typically, TW, PW, RW, and DS were diluted 5-fold and AS were diluted 50-fold. The DOC and DIC concentrations of DW were also measured and subtracted from the final DOC and DIC concentrations.

**Salinity test**. All water samples were filtered with a Whatman quantitative filter paper. The evaporating dishes used for the salinity tests were pre-dried to a constant weight. Approximate 60 mL of filtered samples were added into the evaporating dishes, then evaporated on a water bath. Drops of H_2_O_2_ solution (30%) were added into the evaporating dish until the color of the residue is white. Finally, the evaporating dish was dried in an oven to a constant weight. The test was conducted in duplicates.

**EEM analysis**. PW and DS before and after exposure experiments were filtered (0.45 µm) for the EEM analysis, which was conducted using a VARIAN Cary Eclipse fluorescence spectrophotometer. The water samples were diluted in order to guarantee the maximum emission intensity was lower than the detection limit (1,000 arbitrary units). Typically, PW samples were diluted 5 fold and DS samples were diluted 10 and 50 fold, respectively. EEM spectra were collected with subsequent scanning emission spectra from 250 to 500 nm at 2 nm increments by varying the excitation wavelength from 200 to 450 nm at 10 nm increments. The excitation and emission slit bandwidth were maintained at 5 nm and the scanning speed was set at 1,200 nm/min for all the measurements. The spectrum of DW was recorded as the blank. The EEM data were plotted using Origin 8.0 (OriginLab) with 20 contour lines. The contour intervals were automatically given in the software.


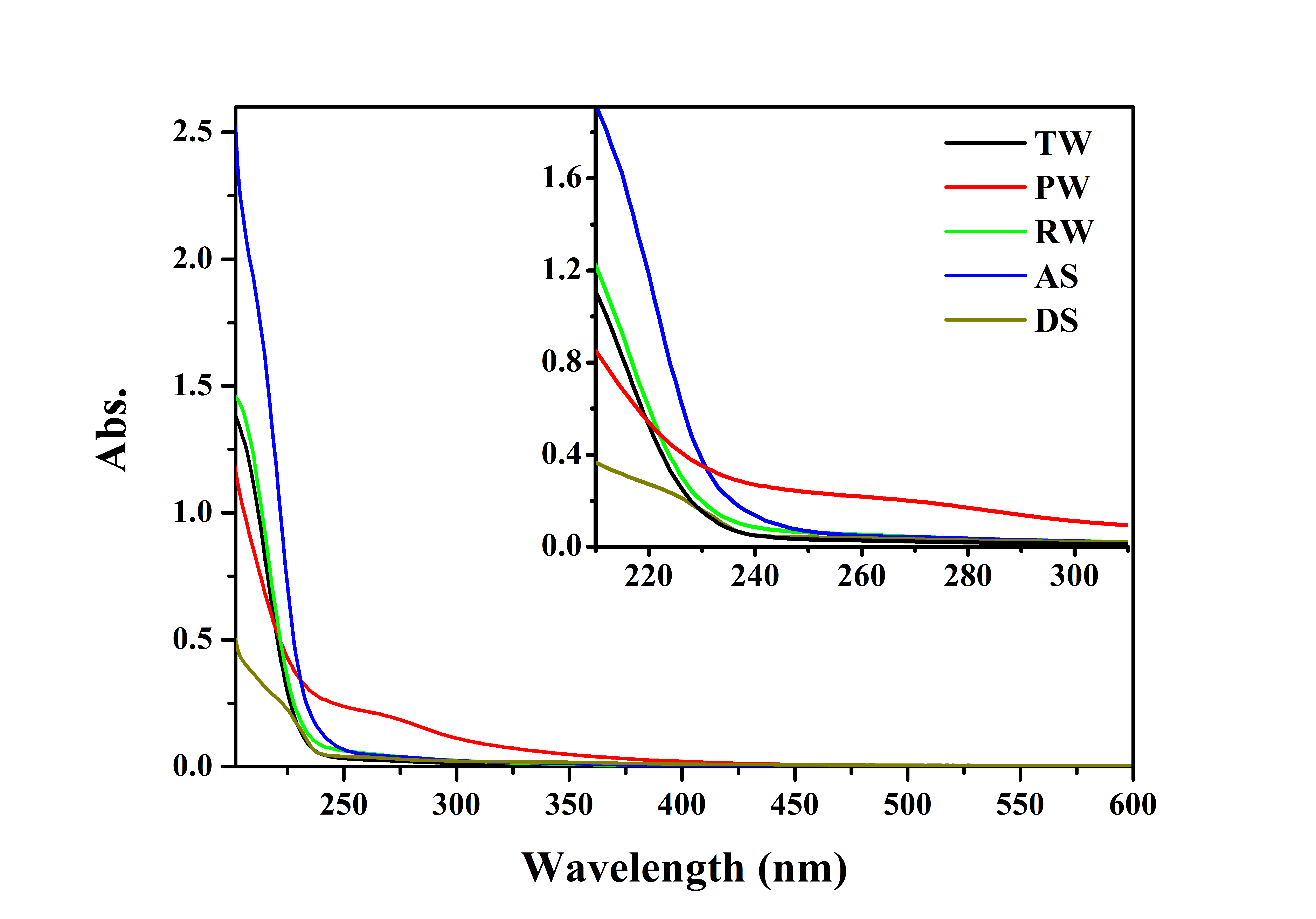


Supplementary Figure S1 UV spectrum of aqueous solutions used in this study. (RW and AS is diluted 5 fold, DS is diluted 10 fold)


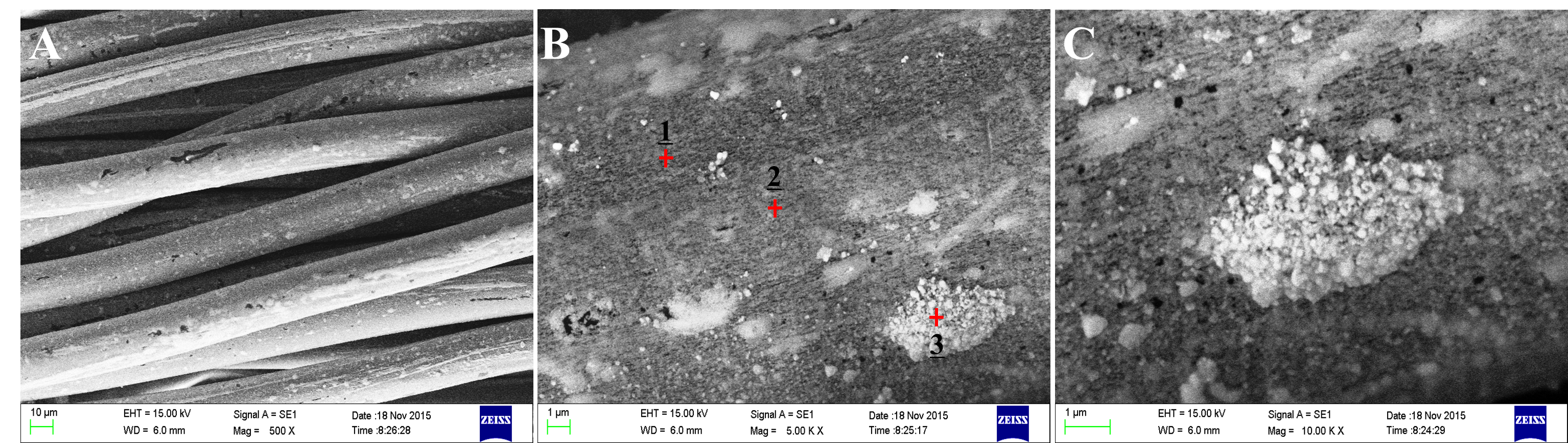


Supplementary Figure S2 The SEM images of aged textile (48 h) after exposure in DW under 35 °C. (A: 500×, B: 5,000×, C: 10,000×)

Supplementary Table S1 EDX results of the selected points in Panel B of Figure S3.

| Point 1 | | | | | Point 2 | | | Point 3 | | |
| --- | --- | --- | --- | --- | --- | --- | --- | --- | --- | --- |
| Element | Mass (%) | | | Atom (%) | Element | Mass (%) | Atom (%) | Element | Mass (%) | Atom (%) |
| C K | | 2.84 | 19.42 | | C K | 2.22 | 15.99 | C K | 1.12 | 9.09 |
| O K | | 0.94 | 4.85 | | O K | 0.67 | 3.65 | Mg K | 0.15 | 0.59 |
| Mg K | | 0.25 | 0.85 | | Mg K | 0.17 | 0.62 | Si K | 0.39 | 1.35 |
| Si K | | 0.46 | 1.36 | | Si K | 0.56 | 1.72 | Ag L | 98.35 | 88.97 |
| Cl K | | 0.43 | 0.99 | | Cl K | 0.42 | 1.03 |  |  |  |
| Se L | | 0.33 | 0.34 | | Ag L | 95.95 | 77.00 |  |  |  |
| Ag L | | 94.75 | 72.19 | |  |  |  |  |  |  |
| Total | | 100.00 |  | | Total | 100.00 |  | Total | 100.00 |  |


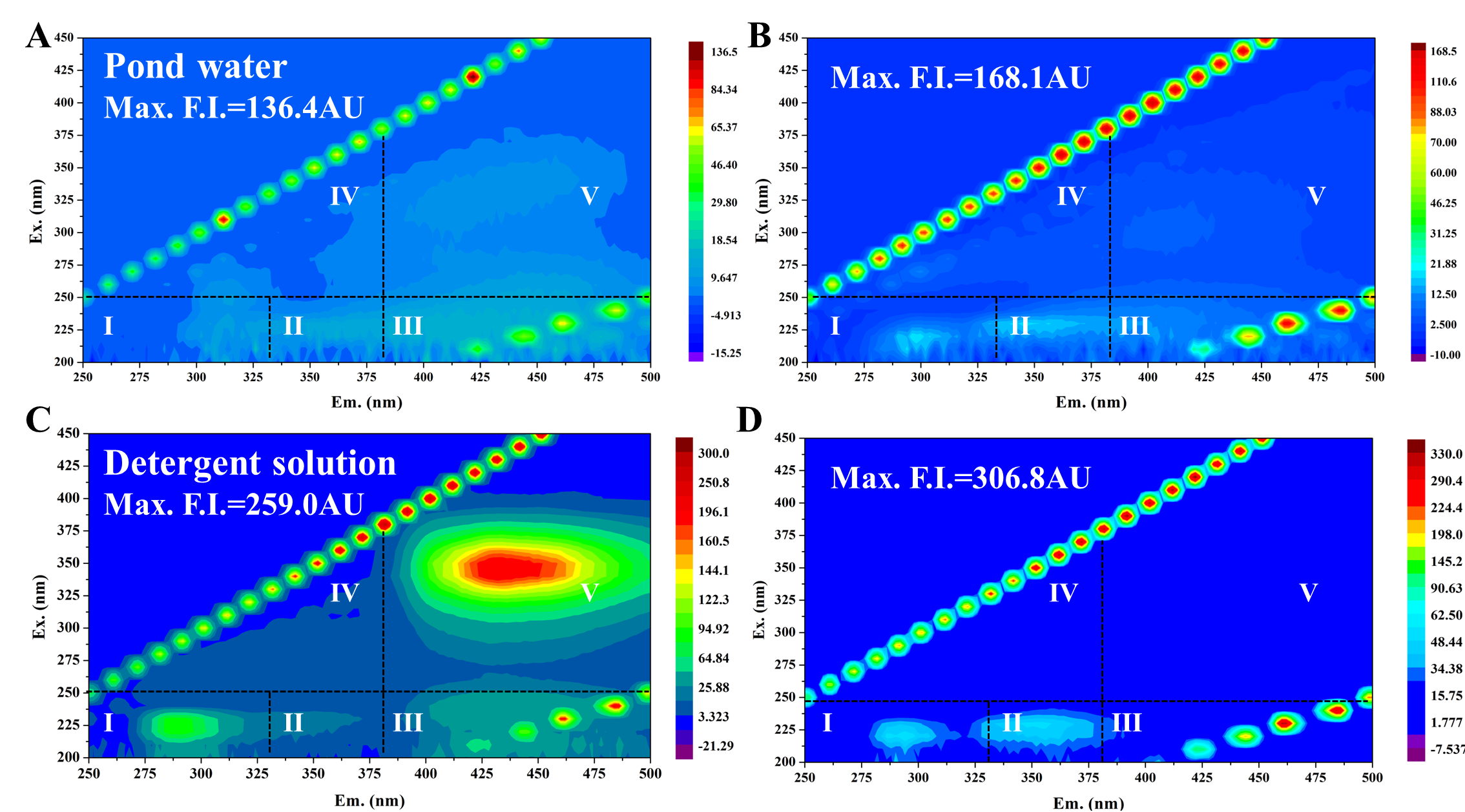


Supplementary Figure S3 EEM spectra of PW and DS before (A and C) and after (B and D) exposure experiments. (B: pristine textile in PW at 25 °C; D: aged textile (48 h) in DS at 25 °C) (Dilution: 5 fold in A and B, 10 fold in C and 50 fold in D)
